# Effectiveness of Antimicrobial Stewardship Program in Long-Term Care: A Five-Year Prospective Single-Center Study

**DOI:** 10.1155/2022/8140429

**Published:** 2022-04-12

**Authors:** Maha Mahmoud Alawi, Wail A Tashkandi, Mohamed A Basheikh, Faten M Warshan, Hazem Ahmed Ghobara, Rosemarie B. Ramos, Mary Leilani Guiriba, Omar Ayob, Safiah Saad Janah, Anees Ahmad Sindi, Suheib Ali Abdulhamid Ahmed, Salah Dammnan, Esam Ibraheem Azhar, Ali A. Rabaan, Salma Alnahdi, Maged Mohammed Bamahakesh

**Affiliations:** ^1^Department of Medical Microbiology and Parasitology, Faculty of Medicine, King Abdulaziz University Hospital, Jeddah, Saudi Arabia; ^2^Infection Control and Environmental Health Unit, King Abdulaziz University Hospital, Jeddah, Saudi Arabia; ^3^Department of Surgery, King Abdulaziz University, Jeddah, Saudi Arabia; ^4^Faculty of Medicine, King Abdulaziz University, Jeddah, Saudi Arabia; ^5^International Extended Care Center, Jeddah, Saudi Arabia; ^6^Infection Control Department, International Extended Care Center, Jeddah, Saudi Arabia; ^7^King Abdulaziz University, Jeddah, Saudi Arabia; ^8^Department of Anesthesia and Critical Care, Faculty of Medicine, King Abdulaziz University, Jeddah, Saudi Arabia; ^9^Special Infectious Agents Unit—BSL3, King Fahd Medical Research Center and Medical Laboratory Technology Department, Faculty of Applied Medical Sciences, King Abdulaziz University, Jeddah, Saudi Arabia; ^10^Molecular Diagnostic Laboratory, Johns Hopkins Aramco Healthcare, Dhahran, Saudi Arabia; ^11^Department of Public Health/Nutrition, The University of Haripur, Haripur, Pakistan

## Abstract

**Objective:**

To report the effectiveness of the antimicrobial stewardship program (ASP) in a long-term care (LTC) facility, by analyzing the change in antimicrobial consumption and cost and multidrug resistance (MDR) rates over a 5-year period.

**Method:**

A prospective interventional study was conducted at a 106-bed facility (nursing home: 100 beds and an intensive care unit (ICU): 6 beds). The ASP was designed and led by a multidisciplinary team including an infectious disease consultant, two clinical pharmacists, a clinical microbiologist, and an infection control preventionist. Five key performance indicators were monitored: (1) intravenous (IV)-to-oral switch rate, (2) consumption of restricted IV antimicrobials (raw consumption and defined daily doses (DDD) index), (3) cost of restricted IV antimicrobials, (4) antimicrobial sensitivity profiles, and (5) MDR rate among hospital-acquired infections (MDR-HAI).

**Result:**

A ∼5.5-fold enhancement of the IV-to-oral switch and a 40% reduction in the overall consumption of restricted IV antimicrobials were observed. Regarding the cost, the cumulative cost saving was estimated as 5.64 million SAR (US$1.50 million). Microbiologically, no significant change in antimicrobial sensitivity profiles was observed; however, a large-size reduction in the MDR-HAI rate was observed, notably in ICU where it declined from 3.22 per 1,000 patient days, in 2015, to 1.14 per 1,000 patient days in 2020. Interestingly, the yearly overall MDR rate was strongly correlated with the level of antimicrobial consumption.

**Conclusion:**

The implementation of a multidisciplinary ASP in LTC facilities should be further encouraged, with emphasis on physicians' education and active involvement to enhance the success of the strategy.

## 1. Introduction

Antimicrobial resistance (AMR) is one of the major challenges to modern healthcare systems, engendering substantial mortality and morbidity, and increasing the economic burden on societies [1–3]. Its association with antibiotics misuse and overuse as a major causative factor elicits the utmost attention of the scientific community and public health authorities [4–7]. Substantial evidence demonstrates a strong correlation between the level and pattern of antimicrobial consumption and prescribing with the development and dissemination of AMR and multidrug-resistant (MDR) organisms [8–10]. Consequently, several interventional programs have been implemented globally to promote the appropriate use of antimicrobials. Antimicrobial stewardship programs (ASPs) are one such intervention program which consists of multidisciplinary coordinative interventions designed to enhance antimicrobial prescribing and use within a given healthcare setting. ASPs achieve remarkable optimization of prescribing practice in a cost-effective manner and aim to minimize the emergence of AMR by reducing the risk of resistant pathogen selection in the long run [11–14].

Antimicrobials consumption is very high in long-term care (LTC) due to the high prevalence of hospital-acquired infections (HAIs) [15–18]. This increases the risk of AMR development, which further compromises the health and prognosis of the vulnerable LTC population [19–21]. Additionally, LTC is evidenced to be serving as a reservoir for resistant pathogens in other healthcare settings [22,23]. Another factor of AMR, which is commonly reported in LTC, is inadequate antibiotic prescribing practice, due to several misconceptions among practitioners and healthcare providers, besides the frequent lack of prescribing guidelines [24–26]. This magnifies the relevance of addressing the issue of AMR in LTC by improving the antimicrobial prescribing practice.

The present study describes and analyzes the effectiveness of ASP that was implemented in an LTC facility in the Western region of Saudi Arabia. Antimicrobial consumption and cost and MDR are among the KPIs that were monitored and analyzed to reflect the effectiveness of the ASP over a 5-year period following its implementation.

## 2. Methods

### 2.1. Design and Setting

This prospective interventional study was approved by the medical board at the International Extended Care Centers. It was conducted at the ICU and LTC at the International Extended Care Centre, Jeddah, Saudi Arabia, between 2015 and 2020. The International Extended Care Centre is a private LTC comprising two units (a 100-bed LTC unit and a 6-bed ICU).

### 2.2. Intervention

#### 2.2.1. Members of the ASP Team

The ASP was implemented at International Extended Care Centers from October 2015 to December 2015. The ASP team was formed and led by an infectious disease consultant and co-led by two clinical pharmacists; all undertook the implementation and supervision of the ASP. Additionally, the team included a clinical microbiologist who was committed to developing and maintaining the hospital antibiogram and optimizing the use of laboratory testing and results reporting. An infection control preventionist was also involved in the team, having the mission to prevent, monitor, and report hospital-acquired infections and resistance.

#### 2.2.2. Mission and Intervention

The ASP intervention was articulated into two broad endeavors including an educational/advisory role and an active role:

(i) The educational/advisory role consisted of the development and implementation of evidence-based empiric anti-infective use guidelines (EAIUG). These guidelines were provided as a manual which was made available as both hard and soft copies, for all prescribers. Several meetings and training sessions involving ASP team members, physicians, and nurses have been held to present the manual and highlight the rationale and importance of the ASP. The list of care situations included in the EAIUG is depicted in Box 1.
(ii) The active role consisted of a set of five actions that were systematically carried out for every antibiotic prescription:
  1. Reviewing the indication by differentiating between empirical prescription and documented infection
  2. Checking the drug appropriateness with regard to the clinical picture (empirical prescription) or culture results
  3. Reviewing the dose, thereby controlling for under- or overdosing, and dose optimization for specific cases such as renal insufficiency, hepatic failure, and resistant pathogens with high minimum inhibitory concentration
  4. Reviewing duration to prevent unnecessarily prolonged duration
  5. Ensuring the implementation of anti-infective use guidelines

The implementation of the active role was carried out using the Antibiotic Stewardship Review and Approval Form (Appendix 1), which was designed specifically for that purpose. The form is filled out and reviewed by the clinical pharmacist and infectious disease consultant.

### 2.3. Key Performance Indicators: Definitions and Scope

Besides the above-mentioned roles, the ASP team undertook reporting and monitoring of the KPIs for the ASP implementation and effectiveness. Six KPIs were initially considered and were defined and reported as follows (Box 2):

1. IV-to-oral switch: it indicates the change in prescribing practice following the ASP implementation. It was defined as the percentage of IV antibiotic prescriptions that were switched to the corresponding oral form in accordance with the specific EAIUG (sheet ^#^23 in Box 1). In the present study, only five index antibiotics were analyzed (ciprofloxacin, levofloxacin, trimethoprim-sulfamethoxazole, and amoxicillin-clavulanate).
2. Consumption of restricted IV antimicrobials: 16 restricted IV antimicrobials included moxifloxacin, levofloxacin, piperacillin, cefepime, colistin, meropenem, imipenem, caspofungin, tigecycline, micafungin, anidulafungin, voriconazole, amphotericin B, linezolid, and amikacin. Consumption was defined and computed as the yearly number of consumed units (vials, boxes, ampoules, or bags) per antibiotic, divided by the corresponding number of patient days. The overall consumption of restricted IV antimicrobials was calculated by pooling the consumed units for all antibiotics.
3. Cost of restricted antibiotics: the number of consumed units was multiplied by the unit cost for each drug, with respect to the applied prices. The cost was calculated and presented as 1,000 Saudi Riyal (kSAR) per 1,000 patient days. Additionally, to adjust for yearly variation in drug prices, adjusted (mean-standardized) costs were calculated using the average price during the six study years (2015–2020).
4. Defined daily dose index: this measure was recommended by the World Health Organization (WHO) as the standard measure of drug utilization, enabling monitoring and comparative analysis of drug consumption across settings and countries [27]. It is defined as “the assumed average maintenance daily dose for a drug used for its main indication in adults”. It is calculated by dividing the actual dose of the drug by the corresponding drug-specific factor, as provided on the WHO official website [28]. In the present study, yearly DDD was calculated per 1,000 patient days. Only ten antibiotics (levofloxacin, piperacillin-tazocilline, cefepime, colistin, meropenem, imipenem, caspofungin, tigecycline, amikacin, and linezolid) were considered for this key performance indicator. Additionally, an overall DDD index was computed as the mean yearly DDD of all antibiotics by 1,000 patient days.
5. Antibiogram: the antibiogram was monitored using semiannual and annual cumulative reports of antimicrobial susceptibility rates of common microbial pathogens to antimicrobials available in the hospital formulary. It was intended to be used as a source for direct empiric antimicrobial therapy. In ASP, the antibiogram was used to monitor the change over time of the antimicrobial susceptibility profile (sensitivity rate) of a given microbial pathogen. Thus, for each year, the number of isolates for each pathogen was used as the denominator to calculate the percentage of isolates that were sensitive to each antimicrobial. Given the number of antimicrobials tested, the present study used the overall antimicrobial sensitivity index for each pathogen, which is an estimate of the overall antimicrobial sensitivity of a given pathogen and is calculated as the average sensitivity rate of the tested antimicrobials. Natural resistance was excluded from this analysis.
6. Multidrug resistance in hospital-acquired infections (MDR-HAI): defined as a pathogen being resistant to at least one agent in three or more antibiotic categories. The yearly number of MDR-HAI was expressed by 1,000 patient days.

### 2.4. Statistical Methods

Data was collected in predesigned Microsoft Excel sheets for each key performance indicator. The sheet was constantly monitored and updated and then closed at the end of each year. The final parameters, such as adjusted costs and adjustment by 1,000 patient days, were computed using calculation functions available in Microsoft Excel. Descriptive statistics were used to present the change in the KPIs over the years. Multifactorial repeated-measures ANOVA (RM-ANOVA) was used to analyze the effect of time (years), unit (intensive care unit versus long-term care), and time^*∗*^unit on the change in the raw number of MDR-HAI, from the start of the ASP in 2015 to 2020. Results are presented as yearly estimated marginal means, Wilk's lambda, and effect size indicated by square Eta. A *p* value of <0.05 was considered statistically significant.

## 3. Results

### 3.1. Effect of the IV-to-Oral Switch

**Figure 1:**
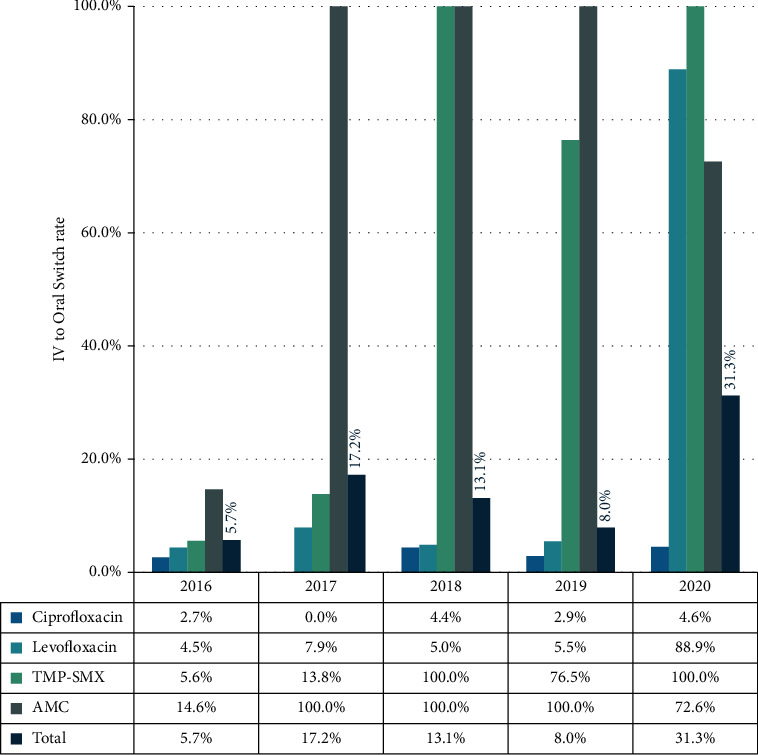
IV-to-oral switch rates of index antibiotics following the implementation of the antimicrobial stewardship program (2016–2020).

Overall, the IV-to-oral switch rate increased from 5.7% in 2016 to 31.3% in 2020 considering all index antibiotics, corresponding to a ∼5.5-fold increase in IV-to-oral switch practice. IV-to-oral switch rate for amoxicillin-clavulanate was 100% from 2017 to 2019 followed by a decline in 2020, while the switch rate for trimethoprim-sulfamethoxazole was 100% in 2018 and 2020 but showed a decrease in 2019 (76.5%). For levofloxacin, a rise in switch rate (88.9%) was observed only in 2020 with reference to 4.5% to 7.9% between 2016 and 2019. The switch rate for ciprofloxacin remained constantly low (0.0%–4.6%) throughout the study period (Figure 1).

### 3.2. Effect on the Consumption of Restricted Antimicrobials

**Figure 2:**
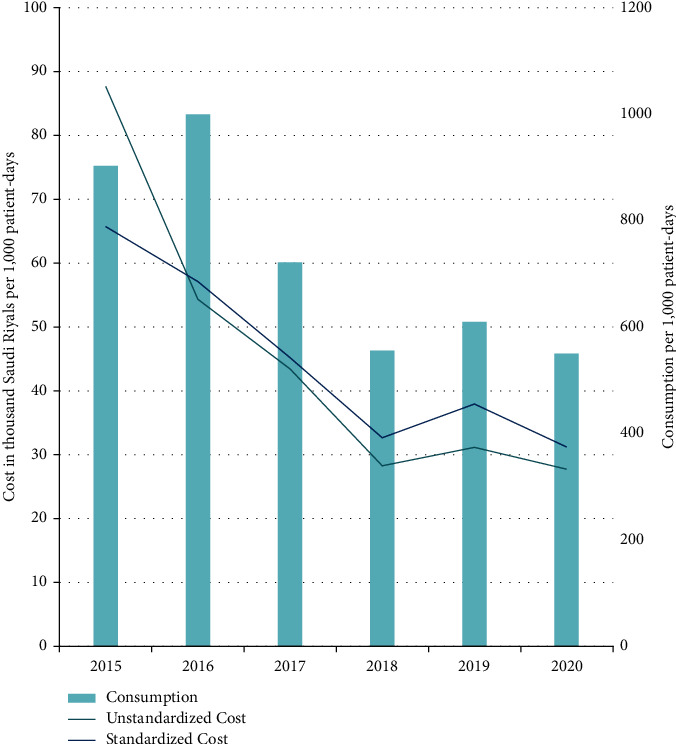
Consumption and cost of restricted injectable antimicrobials per 1,000 patient days, following the implementation of the antimicrobial stewardship program. Standardized costs are calculated by multiplying the actual consumption by the mean prices for each antimicrobial agent. It was used to adjust for the significant variance in cost due to the significant variability of antimicrobials' prices across the years.

After a slight increase from 2015 to 2016 (902.66 to 998.78 doses per 1,000 patient days), the overall consumption of restricted IV antimicrobials decreased to 550.24 doses per 1,000 patient days in 2020, representing a ∼40% decrease in reference to baseline (Figure 2).

### 3.3. Effect on the Cost of Restricted Antimicrobials

**Figure 3:**
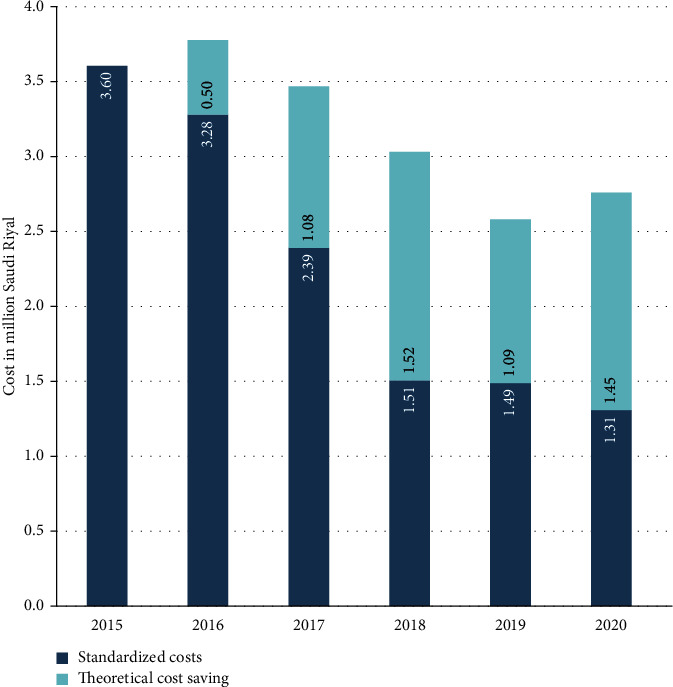
Estimated theoretical cost savings on restricted injectable antimicrobials following the implementation of the antimicrobial stewardship program. Theoretical cost savings for a given year are estimated as the difference between the standardized costs for the year and the standardized costs for baseline (2015) adjusted for the number of patient days of the respective year (2016, 2017,…). This estimate is based on the assumption that, in absence of ASP, the antimicrobial consumption per patient day is consistent, which would result in invariable cost per patient day by using the mean prices for each agent.

The cost of restricted IV antimicrobials was decreased by 38% in 2016, despite increased consumption. This is explained by the significant price decline for several antimicrobials with the advent of the corresponding generic drugs, notably piperacillin-tazobactam 4.5 g (from 33.92 to 24.18 SAR per unit), colistin 2 MIU (from 49.33 to 29.6 SAR), caspofungin 50 mg (from 2250.42 to 709.73 SAR), and caspofungin 70 mg (from 2898.37 to 1450.24 SAR). Afterward, there was a further decline in restricted IV antimicrobials costs reaching 27.66k SAR per 1,000 patient days in 2020, representing a 68% decrease with reference to baseline. Mean-standardized costs of restricted IV antimicrobials showed the same trend with more than 50% cost saving achieved in 2020 with reference to 2015 (Figure 2). By assuming consistent antimicrobial consumption per patient day over the years, and by using the mean price for each agent, the theoretical cumulative cost savings enabled by the ASP over the 5 years are estimated as 5.64 million SAR (US$1.50 million) (Figure 3).

### 3.4. Effect of Defined Daily Doses

**Figure 4:**
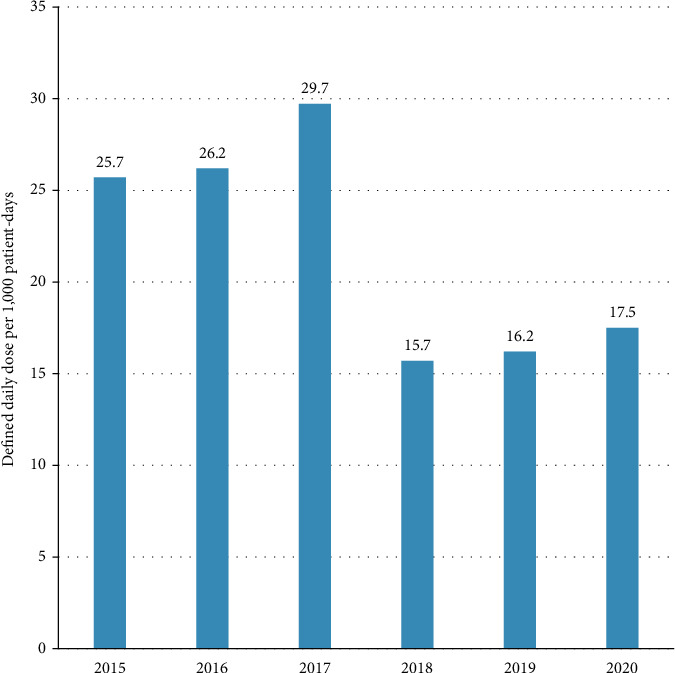
Change is defined as daily doses of restricted antimicrobials per 1,000 patient days following the implementation of the antimicrobial stewardship program.

A decreasing trend of DDD per 1,000 patient days was observed between 2015 and 2019 for most antibiotics except for meropenem which showed a 3-fold increase between 2015 and 2016, and tigecycline which peaked to 116.6 in 2017 then decreased to 3.21 in 2019. In 2020, a reincrease trend in DDD for 5 out of the 10 antibiotics was observed (Table 1). The average DDD of all antibiotics showed a two-phase figure, including an initial high phase (25.7–29.7 per 1,000 patient days) between 2015 and 2017, followed by a low phase (15.7–17.5 per 1,000 patient days) between 2018 and 2020, corresponding to ∼40% decrease (Figure 4).

### 3.5. Effect on Antimicrobial Sensitivity

**Figure 5:**
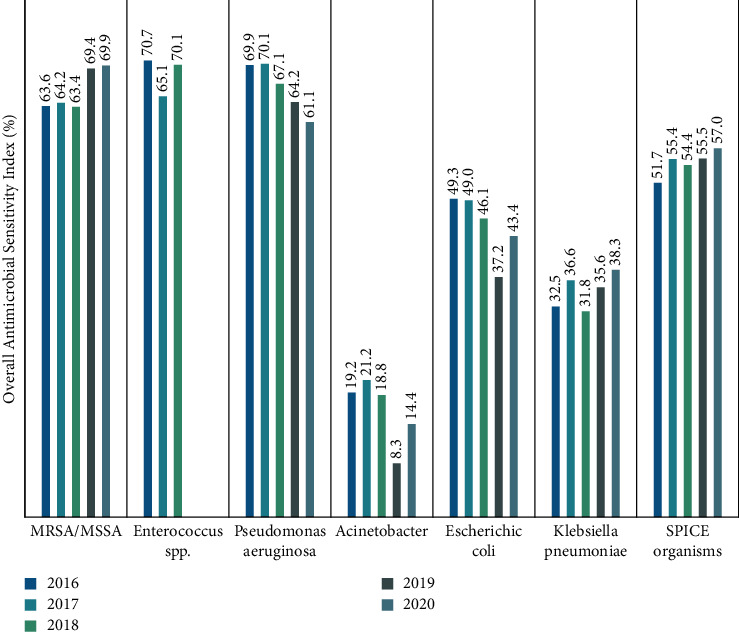
Overall antimicrobial sensitivity index (OASI) of different microorganisms between 2016 and 2020. OASI: overall antimicrobial sensitivity index is an estimate of the sensitivity of a microorganism to the different antimicrobials and is calculated as the average percentage of sensitive isolates in the tested antimicrobials.

There was no significant change in the overall sensitivity indices for the majority of the assessed microorganisms (22 tested antimicrobials) between 2016 and 2020. Nevertheless, a relative increase was observed in the overall antimicrobial sensitivity index of methicillin-resistant *Staphylococcus aureus*/methicillin-susceptible *Staphylococcus aureus* (MRSA/MSSA) (from 63.6% to 69.9%), *K. pneumonia* (32.5% to 38.3%), and SPICE organisms (51.7% to 57.0%) from baseline to 2020, respectively (Figure 5). SPICE stands for *Serratia*, *Providencia*, “Indole-positive” (*Proteus*, *Morganella*, *Providencia*) species/*Acinetobacter*, *Citrobacter*, and *Enterobacter* species.

### 3.6. Effect on Multidrug Resistance: Hospital-Acquired Infections

**Figure 6:**
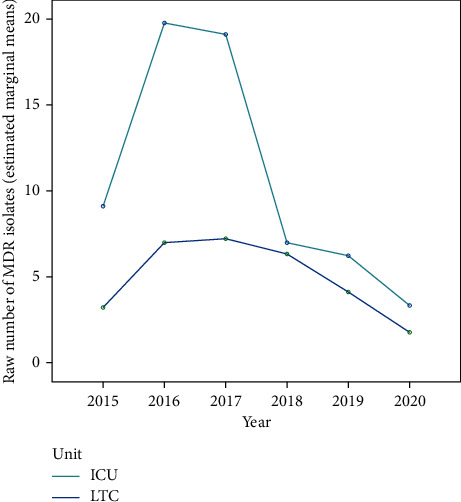
Estimated marginal means of the number of isolated multidrug-resistant (MDR) organisms in hospital-acquired infections, in intensive care (ICU) and long-term care (LTC) units, during the five years following the implementation of the antimicrobial stewardship program.

The number of MDR-HAI showed an inverted U-shaped curve in both ICU and LTC, with an initial increase in 2016–2017, followed by a decrease to reach the lowest rates in 2020 (Figure 6). Multifactorial repeated-measures ANOVA showed a significant effect of time on the variability of the number of isolated MDR-HAI, explaining 68.9% of its variability, respectively, with a very large effect. On the other hand, the effect of the unit was not significant (*p*=0.071) (Table 2).

**Figure 7:**
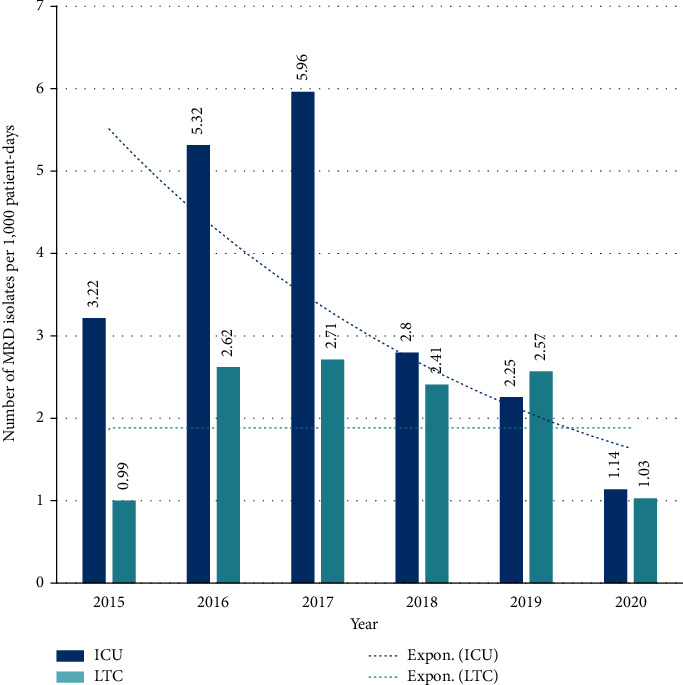
Progression of the number of isolated multidrug-resistant (MDR) organisms in hospital-acquired infections per 1,000 patient days in intensive care (ICU) and long-term care (LTC) units, during the five years following the implementation of the antimicrobial stewardship program.

The number of MDR isolates in HAI per 1,000 patient days showed an inverted U-shaped curve in both ICU and LTC (Figure 7). Of note, subsequent to the initial increase, MDR rates reached lower levels in ICU in 2020 than the rate in 2015 (baseline) (from 3.22 to 1.14 per 1,000 patient days) whereas they reached similar levels as the baseline in LTC (0.99 to 1.03 per 1,000 patient days).

Furthermore, the yearly overall MDR rate was highly correlated with the yearly raw antimicrobial consumption, with a Pearson's correlation coefficient as high as 0.941 (*p*=0.005).

## 4. Discussion

### 4.1. Summary of Findings

This 5-year prospective study showed the effectiveness of ASP in improving antimicrobial prescribing practice in an LTC facility while reducing the overall consumption of both IV and oral antimicrobials. Compared with baseline, the intervention enabled a ∼5.5-fold enhancement of IV-to-oral switch and a 40% reduction in the overall antibiotic use (both raw consumption and the WHO recommended DDD index). This resulted in an approximately 60% savings on the actual costs of restricted antimicrobials and an estimated theoretical cost saving of 5.64 million SAR (US$1.50 million). Furthermore, a reduction in antimicrobial consumption was associated with a large-size reduction in the MDR-HAI rate, notably in ICU where it was reduced by∼65% with reference to a baseline, and the yearly overall MDR rate was strongly correlated with the level of antimicrobial consumption.

### 4.2. Antimicrobial Stewardship Program Improved Prescribing Practice

Prior to the ASP implementation, antimicrobial use was characterized by prolonged IV courses with inadequate practice to the switch to oral form; the switch rate was very low and the choice of the oral form was inappropriate in several cases. The ASP prompted the switch to practice while providing a guide for optimal selection of the oral form with respect to the IV drug and the patient's clinical state. However, this effect was delayed in the 2nd year, i.e., 2017, where a 100% switch rate was observed for amoxicillin-clavulanate. Unfortunately, prior data on IV-to-oral switch was not available for comparison, due to the absence of such practice before the ASP implementation. From 2018 to 2020, the total switch rate increased from 13.1% to 31.3%, due to improved switch practice for levofloxacin and trimethoprim-sulfamethoxazole, besides amoxicillin-clavulanate. However, a decline in switch practice for amoxicillin-clavulanate in 2020 was observed, which may be attributed to the COVID-19 outbreak in the facility that required prolonged IV treatment for several patients [29].

The other aspect of improved antimicrobial prescribing practice was the substantial decrease in overall consumption of restricted antimicrobials, reaching ∼40% by 2020 with reference to 2015 (baseline). The transient increase in consumption observed in 2016 may be partially due to the increased use of antimicrobials that require longer treatment durations, such as colistin [30]. Indeed, a shift in the prescribing pattern was observed in 2016 favoring colistin, meropenem, amikacin, amphotericin B, and anidulafungin over levofloxacin, cefepime, imipenem, tigecycline, and micafungin. This shift in prescribing pattern also explains the decrease in the cost in 2016, despite the increase in consumption, as discussed in the subsequent section. The change in antimicrobial consumption was further demonstrated by the 40% decrease in 2020 DDD per 1,000 patient days.

Educating the prescribers on the optimal use of antimicrobials is a key objective of ASP and a critical success factor, notably in the long term. While prescribers attempt to offer the optimal treatment when dealing with a single case of their patient, they should remain committed to the related public health issue of preserving the efficacy of antibiotics and preventing the emergence of AMR [31]. Due to several factors, this dual responsibility may be challenging in routine practice, which results in qualitative or quantitative misuse of antibiotics [32,33]. In line with our findings, a study by Cisneros et al. showed that the implementation of ASP in a tertiary care center was followed by a reduction of inappropriate antimicrobial prescriptions from 53.0% to 26.4%, along with a 32% reduction of DDD per 1,000 occupied beds-days [34]. Another UK national study compared the antimicrobial prescribing practice before and after the implementation of the Quality Premium, a financially rewarding program that was launched in 2015, aimed at reducing antimicrobial consumption in primary care. Results showed a 5.4% decrease in antimicrobials prescribed during the first year of the program, accounting for ∼2 million fewer items dispensed, along with an 18.5% decrease in broad-spectrum antibiotics dispensation. Two years later, further analysis showed the sustained effects of the program [35]. Such observations stress the importance of the educational dimension of the ASP to optimize the antimicrobial prescribing practice and enable its sustained effect.

Another aspect of the improved practice is the microbiologically targeted therapy. Although the present study did not analyze this indicator per se, the active role of the ASP team in reviewing and assisting antimicrobial prescriptions has indisputably enhanced the optimization of the prescribing practice in compatibility with microbiology and antimicrobial sensitivity tests. This explains the previously discussed shift in antimicrobial prescribing patterns observed in 2016. A study by Katsios et al. [36] demonstrated a ∼35% reduction in the treatment of patients with nonsterile-site cultures versus a ∼30% increase in the treatment of those sterile-site cultures after the implementation of ASP. Furthermore, the authors observed an improvement in other aspects of the prescribing practices, notably a ∼2.7-fold increase in antimicrobial regimen documentation along with a significant increase in the documentation of antimicrobial duration. However, the success of the ASP strategy in promoting microbiologically optimized antimicrobial prescribing requires timeliness and availability of microbiology tests and results [37].

### 4.3. Antimicrobial Stewardship Program Enabled Considerable Cost Savings

The implementation of ASP was followed by an early significant decline in antimicrobial costs, despite the increase in consumption in 2016. This is probably a positive effect of the qualitative improvement of antimicrobial prescribing led by the ASP team. Afterward, a rapid decline was observed in adjusted costs until 2018, as an effect of the decline in antimicrobial consumption, which was followed by a plateau between 2018 and 2020. As of 2020, the adjusted costs per patient day decreased by more than 50%, for overall cost savings estimated at US$1.50 million, by assuming fixed prices of the drugs, representing a yearly average of US$300,000. A comparable figure was reported in a community hospital, where the implementation of an ASP enabled a 14% reduction in costs during the first year, representing US$228,911 [38]. ASPs are reputed to be highly cost-effective, which may vary by region, facility size, and level of baseline use/misuse of antimicrobials, as well as the method used to calculate the costs. A systematic review including 146 studies from all continents showed a significant reduction in antimicrobial expenditure in 92% of the studies following ASP implementation, with cost savings reaching up to 80%. Besides antimicrobial expenditure, several studies considered other indirect costs in estimating the ASP cost-effectiveness, such as operational and implementation costs and costs associated with the length of hospital stay. This resulted in an average overall cost savings of US$435,000 per year per hospital, with great regional variability [39]. In the present study, the authors estimated only cost savings on antimicrobial consumption, as the overall cost-effectiveness of the intervention was beyond the scope of the study.

### 4.4. Antimicrobial Stewardship Program to Reduce Antimicrobial Resistance and Multidrug Resistance

Probably, the most impactful effect of ASP is the reduction of the MDR-HAI rate, which was achieved with a 3-year lag and was more remarkable in the ICU where it accounted for a 65% decrease as of 2020 with reference to 2015. This reduction was preceded by an initial resurgence of MDR with an uncertain relationship with ASP. On the other hand, a relatively unchanging antimicrobial sensitivity rate of the seven pathogens studied was observed, as demonstrated by the overall antimicrobial sensitivity index. Notwithstanding the difficult interpretation of these findings, profuse evidence demonstrates the correlation between antimicrobial misuse and overuse with the emergence of AMR and MDR strains. A European study involving 29 countries showed a strong to moderate correlation between antimicrobial consumption and AMR, with the highest significance for methicillin-resistant *Staphylococcus aureus* (MRSA), *Klebsiella pneumoniae* resistant to carbapenems, *Streptococcus pneumoniae* resistant to macrolides, and *Escherichia coli* resistant to fluoroquinolones, aminopenicillins, or carbapenems.9 A mathematical model demonstrated the influence of antimicrobial consumption on AMR, highlighting several “antibiotic/resistant pathogen” pairs that enable predicting the change in resistance rate as a function of antibiotic consumption. Consistent with these reports and despite being underpowered as an analysis, the present study showed a strong positive correlation between yearly MDR-AHI rates and raw antimicrobial consumption. As a consequence, it can be hypothesized that the reduction in the MDR rate observed in the present study may be the effect of the qualitative and quantitative optimization of antimicrobial prescribing and use, which was achieved through the combined educational and restrictive methods of ASP. A review including 17 studies showed that different ASP protocols enabled a significant reduction in AMR and MDR, such as extended-spectrum beta-lactamase (ESBL) producing *E. coli* and *Klebsiella*, *Clostridium difficile*, etc. [40]. A single-center study from India showed a 4.5% to 41% reduction in the rate of resistant pathogens, notably ESBL, *E. coli,* and Klebsiella, and carbapenem-resistant *Pseudomonas* as an effect of ASP [41]. Besides the significant benefit on morbidity and mortality, controlling AMR and MDR has an additional cost-saving benefit, with an estimated US$7,300 cost savings and US$9,800 cost-per-life-year for every avoided AMR [42]. This further demonstrates the potential impact of a well-conducted ASP.

### 4.5. Limitations

The major limitations of this study are the single-center design and the absence of some relevant data from the pre-ASP phase. Additionally, the initial phase of ASP was marked by a refractory attitude of physicians, notably in the LTC unit, which challenged the change of prescribing habits and the implementation and adherence to the EAIUG.

## 5. Conclusion

The implementation of a multidisciplinary ASP in LTC facilities considerably improved antimicrobial prescribing practice, both quantitatively and qualitatively. This resulted in an early reduction of antimicrobial expenditure and considerable cumulative cost savings estimated as 5.64 million SAR (US$1.50 million). Microbiologically, a large-size reduction in the MDR-HAI rate was observed with a 3-year lag from the start of the ASP and was strongly correlated with the level of antimicrobial consumption, suggesting a delayed effect of ASP. The authors strongly recommend ASP implementation in LTC facilities, with emphasis on physicians' education and active involvement to enhance the success of the strategy.

## Figures and Tables

**Table 1:** Change in antibiotics defined daily doses per 1,000 patient days following the implementation of the antimicrobial stewardship program.

Antibiotic	Defined daily dose per 1,000 patient days					
	2015	2016	2017	2018	2019	2020
Meropenem	26.1	79.9	71.8	82.7	80.3	92.0
Imipenem	46.4	33.5	14.3	0.0	0.0	0.0
Piperacillin/Tazobactam	67.5	64.8	27.5	25.7	35.5	46.2
Colistin	24.6	34.6	22.0	19.5	15.1	15.5
Caspofungin	12.3	4.4	15.1	9.5	14.6	1.4
Levofloxacin	24.6	5.2	3.3	4.1	1.3	0.9
Linezolid	5.1	3.2	3.6	2.9	0.5	0.6
Amikacin	19.2	19.3	14.6	10.2	9.5	13.6
Tigecycline	20.4	6.6	116.6	2.2	3.2	1.7
Cefepime	10.6	10.2	8.5	0.2	2.0	2.7
Overall (average)	25.7	26.2	29.7	15.7	16.2	17.5

**Table 2:** Effect of time and unit on the number of isolated multidrug-resistant organisms in hospital-acquired infections from 2015 to 2020.

Effect	Wilk's lambda	*p* value	Effect size (squared Eta)	Interpretation
Time (year)	0.311	0.008^*∗*^	0.689	The effect of time explained 68.9% of the variability in the number of isolated MDRs in HAI
Unit	-	0.071	0.189	The effect of the unit alone was not significant to explain the variability in the number of isolated MDRs in HAI
Time^*∗*^unit	0.409	0.036^*∗*^	0.591	The effect of the time^*∗*^unit explained 59.1% of the variability in the number of isolated MDRs in HAI

MDR: multidrug-resistant; HAI: hospital-acquired infections; Multifactorial Repeated-Measure ANOVA analyzing the effect of time (year) and unit (intensive care unit versus long-term care) on the change in the raw number of multidrug-resistant organisms isolated in hospital-acquired infections, from the start of the antimicrobial stewardship program in 2015 to 2020. The yearly estimated marginal means are depicted by the unit in Figure 6. Squared Eta >0.14 indicates a large effect. ^*∗*^Statistically significant result (*p* < 0.05).

## Data Availability

Data supporting this study are available upon a written, motivated request to the author.

## Supplementary Materials

Supplementary Materials Box 1: the content of the evidence-based empiric anti-infective use guidelines (EAIUG) implemented at the International Extended Care Centre (IECC), Jeddah, Saudi Arabia. Description: the box contains guidelines covering 26 care situations related to infectious disease Box 2: key performance indicators (KPIs) of the antimicrobial stewardship program—Definition and Scope. Description: the study used 6 key performance indicators. Appendix 1: Antimicrobial Stewardship Review and Approval Form. Description: The Antibiotic Stewardship Review and Approval Form is filled and reviewed by the clinical pharmacist and infectious disease consultant. The form comprises 4 sections including (a) patient identification (name, gender, hospital number, location, and date), (b) the antibiotic requested (name, dose, route, frequency, duration), (c) the indication and management plan including whether it was prescribed empirically and whether appropriate investigations have been carried out, review plan, and diagnosis, and (d) the approval section including the MRP and Clinical Pharmacist identifications and date of approval. The implementation of the active role was carried out using the Antibiotic Stewardship Review and Approval Form, which was designed specifically for that purpose. It is filled and reviewed by the clinical pharmacist and infectious disease consultant. The form comprises 4 sections including (a) patient identification (name, gender, hospital number, location, and date), (b) the antibiotic requested (name, dose, route, frequency, duration), (c) the indication and management plan including whether it was prescribed empirically and whether appropriate investigations have been carried out, review plan, and diagnosis, and (d) the approval section including the MRP and Clinical Pharmacist identifications, and date of approval.

## References

[1] Dadgostar P. (2019). Antimicrobial resistance: implications and costs. *Infection and Drug Resistance*.

[2] Cassini A., Högberg L. D., Plachouras D. (2019). Attributable deaths and disability-adjusted life-years caused by infections with antibiotic-resistant bacteria in the EU and the European economic area in 2015: a population-level modelling analysis. *Lancet Infectious Diseases*.

[3] Hofer U. (2019). The cost of antimicrobial resistance. *Nature Reviews Microbiology*.

[4] Prestinaci F., Pezzotti P., Pantosti A. (2015). Antimicrobial Resistance: A Global Multifaceted Phenomenon. *Pathogens and Global Health*.

[5] Cao J., Song W., Gu B. (2013). Correlation between carbapenem consumption and antimicrobial resistance rates of acinetobacter baumannii in a university-affiliated hospital in China. *Journal of Clinical Pharmacology*.

[6] Medina E., Pieper D. H. (2016). Tackling threats and future problems of multidrug-resistant bacteria. *Current Topics in Microbiology and Immunology*.

[7] Karam G., Chastre J., Wilcox M. H., Vincent J. L. (2016). Antibiotic strategies in the era of multidrug resistanc. *Critical Care*.

[8] Chaouch C., Hassairi A., Riba M., Boujaafar N. (2014). Relations entre la résistance bactérienne et la consommation des antibiotiques. *Annales de Biologie Clinique*.

[9] McDonnell L., Armstrong D., Ashworth M., Dregan A., Malik U., White P. (2017). National disparities in the relationship between antimicrobial resistance and antimicrobial consumption in Europe: an observational study in 29 Countries. *Journal of Antimicrobial Chemotherapy*.

[10] Arepyeva M. A., Kolbin A. S., Sidorenko S. V. (2017). A mathematical model for predicting the development of bacterial resistance based on the relationship between the level of antimicrobial resistance and the volume of antibiotic consumption. *Journal of Global Antimicrobial Resistance*.

[11] Garau J., Bassetti M. (2018). Role of pharmacists in antimicrobial stewardship programmes. *International Journal of Clinical Pharmacy*.

[12] Molina J., Peñalva G., Gil-Navarro M. V. (2017). Long-term impact of an educational antimicrobial stewardship program on hospital-acquired candidemia and multidrug-resistant bloodstream infections: a quasi-experimental study of interrupted time-series analysis. *Clinical Infectious Diseases*.

[13] Manning M. L., Septimus E. J., Ashley E. S. D. (2018). Antimicrobial stewardship and infection prevention—leveraging the synergy: a position paper update. *American Journal of Infection Control*.

[14] Alawi M. M., Darwesh B. M. (2016). A stepwise introduction of a successful antimicrobial stewardship program: experience from a tertiary care university hospital in western, Saudi Arabia. *Saudi Medical Journal*.

[15] Cotter M., Donlon S., Roche F., Byrne H., Fitzpatrick F. (2012). Healthcare-associated infection in Irish long-term care facilities: results from the First National Prevalence Study. *Journal of Hospital Infection*.

[16] Heudorf U., Boehlcke K., Schade M. (2012). Healthcare-associated infections in long-term care facilities (HALT) in frankfurt am main, Germany, january to march 2011. *European Communicable Disease Bulletin*.

[17] Wójkowska-Mach J., Gryglewska B., Czekaj J., Adamski P., Grodzicki T., Heczko P. B. (2013). Infection control: point prevalence study versus incidence study in polish long-term care facilities in 2009-2010 in the Małopolska Region. *Infection*.

[18] Moro M. L., Ricchizzi E., Morsillo F. (2013). Infections and antimicrobial resistance in long term care facilities: a national prevalence study. *Annali Di Igiene: Medicina Preventiva e Di Comunita*.

[19] Nicolle L. E. (2014). Infection prevention issues in long-term care. *Current Opinion in Infectious Diseases*.

[20] van Buul L. W., van der Steen J. T., Veenhuizen R. B. (2012). Antibiotic use and resistance in long term care facilities. *Journal of the American Medical Directors Association*.

[21] O’Fallon E., Pop-Vicas A., D’Agata E. (2009). The emerging threat of multidrug-resistant gram-negative organisms in long-term care facilities. *The Journals of Gerontology Series A: Biological Sciences and Medical Sciences*.

[22] van den Dool C., Haenen A., Leenstra T., Wallinga J. (2016). The role of nursing homes in the spread of antimicrobial resistance over the healthcare network. *Infection Control & Hospital Epidemiology*.

[23] Verhoef L., Roukens M., De Greeff S., Meessen N., Natsch S., Stobberingh E. (2016). Carriage of antimicrobial-resistant commensal bacteria in Dutch long-term-care facilitie. *Journal of Antimicrobial Chemotherapy*.

[24] Ricchizzi E., Latour K., Kärki T. (2018). Antimicrobial use in european long-term care facilities: results from the third point prevalence survey of healthcare-associated infections and antimicrobial use, 2016 to 2017. *Euro Surveillance*.

[25] Fleming A., Bradley C., Cullinan S., Byrne S. (2014). Antibiotic prescribing in long-term care facilities: a qualitative, multidisciplinary investigation. *BMJ Open*.

[26] Lim C. J., Stuart R. L., Kong D. C. (2015). Antibiotic use in residential aged care facilities. *Australian Family Physician*.

[27] World Health Organization (2021). Defined daily dose (DDD). https://www.who.int/tools/atc-ddd-toolkit/about-ddd.

[28] World Health Organization (2021). ATC/DDD Index. https://www.whocc.no/atc_ddd_index/.

[29] Alawi M. M. S. (2021). Successful management of COVID-19 outbreak in a long-term care facility in Jeddah, Saudi Arabia: epidemiology, challenges for prevention and adaptive management strategies. *Journal of Infection and Public Health*.

[30] Markou N., Apostolakos H., Koumoudiou C. (2003). Intravenous colistin in the treatment of sepsis from multiresistant gram-negative bacilli in critically Ill patients. *Critical Care*.

[31] Pulcini C., Gyssens I. C. (2013). How to educate prescribers in antimicrobial stewardship practices. *Virulence*.

[32] Alnemri A. R., Almaghrabi R. H., Alonazi N., Alfrayh A. R. (2016). Misuse of antibiotic: a systemic review of Saudi published studies. *Current Pediatric Research*.

[33] Calbo E., Álvarez-Rocha L., Gudiol F., Pasquau J. (2013). A review of the factors influencing antimicrobial prescribing. *Enfermedades Infecciosas Y Microbiología Clínica*.

[34] Cisneros J. M., Neth O., Gil-Navarro M. V. (2014). Global impact of an educational antimicrobial stewardship programme on prescribing practice in a tertiary hospital centre. *Clinical Microbiology and Infections*.

[35] Balinskaite V., Johnson A. P., Holmes A., Aylin P. (2019). The impact of a national antimicrobial stewardship program on antibiotic prescribing in primary care: an interrupted time series analysis. *Clinical Infectious Diseases*.

[36] Katsios C. M., Burry L., Nelson S. (2012). An antimicrobial stewardship program improves antimicrobial treatment by culture site and the quality of antimicrobial prescribing in critically Ill patients. *Critical Care*.

[37] Skodvin B., Aase K., Charani E., Holmes A., Smith I. (2015). An antimicrobial stewardship program initiative: a qualitative study on prescribing practices among hospital doctors. *Antimicrobial Resistance and Infection Control*.

[38] Malani A. N., Richards P. G., Kapila S., Otto M. H., Czerwinski J., Singal B. (2013). Clinical and economic outcomes from a community hospital’s antimicrobial stewardship program. *American Journal of Infection Control*.

[39] Nathwani D., Varghese D., Stephens J., Ansari W., Martin S., Charbonneau C. (2019). Value of hospital antimicrobial stewardship programs [ASPs]: a systematic review. *Antimicrobial Resistance and Infection Control*.

[40] Goff D. A., File T. M. (2016). The evolving role of antimicrobial stewardship in management of multidrug resistant infections. *Infectious Disease Clinics of North America*.

[41] Jaggi N., Sissodia P., Sharma L. (2012). Control of multidrug resistant bacteria in a tertiary care hospital in India. *Antimicrobial Resistance and Infection Control*.

[42] Ruiz-Ramos J., Frasquet J., Romá E. (2017). Cost-effectiveness analysis of implementing an antimicrobial stewardship program in critical care units. *Journal of Medical Economics*.

