# Supplementary material for: Effectiveness of Antimicrobial Stewardship Program in Long-Term Care: A Five-Year Prospective Single-Center Study
Source: Interdiscip Perspect Infect Dis. 2022 Apr 12;2022:8140429. doi: 10.1155/2022/8140429 (PMC9019452; doi:10.1155/2022/8140429)
Supplement: Supplementary Materials — Box 1: the content of the evidence-based empiric anti-infective use guidelines (EAIUG) implemented at the International Extended Care Centre (IECC), Jeddah, Saudi Arabia. Description: the box contains guidelines covering 26 care situations related to infectious disease Box 2: key performance indicators (KPIs) of the antimicrobial stewardship program—Definition and Scope. Description: the study used 6 key performance indicators. Appendix 1: Antimicrobial Stewardship Review and Approval Form. Description: The Antibiotic Stewardship Review and Approval Form is filled and reviewed by the clinical pharmacist and infectious disease consultant. The form comprises 4 sections including (a) patient identification (name, gender, hospital number, location, and date), (b) the antibiotic requested (name, dose, route, frequency, duration), (c) the indication and management plan including whether it was prescribed empirically and whether appropriate investigations have been carried out, review plan, and diagnosis, and (d) the approval section including the MRP and Clinical Pharmacist identifications and date of approval. The implementation of the active role was carried out using the Antibiotic Stewardship Review and Approval Form, which was designed specifically for that purpose. It is filled and reviewed by the clinical pharmacist and infectious disease consultant. The form comprises 4 sections including (a) patient identification (name, gender, hospital number, location, and date), (b) the antibiotic requested (name, dose, route, frequency, duration), (c) the indication and management plan including whether it was prescribed empirically and whether appropriate investigations have been carried out, review plan, and diagnosis, and (d) the approval section including the MRP and Clinical Pharmacist identifications, and date of approval. [file 8140429.f1.zip › 8140429.f1/Appendix 1 - ASP Approval Form.pdf]

IEC
المركز الدولي للرعاية الممتدة
International Extended Care Centre

بالقلب نرعاكم
We embrace, we care

Antibiotic Stewardship Review & Approval Form

Patient Name ______________________________ **Patient MRN** ____________________

Sex: ☐ Female ☐Male **Location:** ☐ B2, ☐ B3, ☐ B4. **Date:** / / 2020

Antibiotic Requested: ☐ Moxifloxacin ☐ Levofloxacin ☐ Piperacillin/Tazobactam ☐ Cefepime ☐ Colistin

☐ Meropenem ☐ Imipenem ☐ Caspofungin ☐ Tigecycline

☐ Micafungin ☐ Anidulafungin ☐ Voriconazole ☐ Amphotericin ☐ Linezolid ☐ Amikacin ☐ Acyclovir

Dosage Form: ☐ I.V. ☐ Nebulization ☐ Oral

Dose: ____________ Frequency ______________ Duration:____________ CrCl: ____________ ☐ Dialysis

Indication:

a) **Empirical ☐:** ____

- **Appropriate investigations were obtained:** ☐ Peripheral blood culture ☐Central blood culture ☐ Urine analysis

☐Urine culture ☐ Sputum ☐ Wound ☐Stool for toxin ☐CSF ☐ Chest-X-ray ☐CT

b) **To be reviewed in_______ Days**

C) **Therapeutic ☐**:

- ☐ HAP ☐VAP ☐ Bacteremia ☐UTI ☐Pyelonephritis ☐Candidemia

☐Osteomylities ☐Wound infection ☐ Cellulites ☐ Meningitis ☐ Line Infection

☐ Shunt ☐ Other: _________

Diagnosis:____

Plan:____

____

MRP Name: ____________________ **Signature:** ______________________ **Date:** _______________

Clinical Pharmacist Name: ____________________ **Signature:** ________________ **Date:** ___________

Created By: Maha.M.Alawi, MD, FRCPC, CIC
Revised By: Pharmacy Department Jan, 2020
