# Supplementary material for: Effectiveness of Antimicrobial Stewardship Program in Long-Term Care: A Five-Year Prospective Single-Center Study
Source: Interdiscip Perspect Infect Dis. 2022 Apr 12;2022:8140429. doi: 10.1155/2022/8140429 (PMC9019452; doi:10.1155/2022/8140429)
Supplement: Supplementary Materials — Box 1: the content of the evidence-based empiric anti-infective use guidelines (EAIUG) implemented at the International Extended Care Centre (IECC), Jeddah, Saudi Arabia. Description: the box contains guidelines covering 26 care situations related to infectious disease Box 2: key performance indicators (KPIs) of the antimicrobial stewardship program—Definition and Scope. Description: the study used 6 key performance indicators. Appendix 1: Antimicrobial Stewardship Review and Approval Form. Description: The Antibiotic Stewardship Review and Approval Form is filled and reviewed by the clinical pharmacist and infectious disease consultant. The form comprises 4 sections including (a) patient identification (name, gender, hospital number, location, and date), (b) the antibiotic requested (name, dose, route, frequency, duration), (c) the indication and management plan including whether it was prescribed empirically and whether appropriate investigations have been carried out, review plan, and diagnosis, and (d) the approval section including the MRP and Clinical Pharmacist identifications and date of approval. The implementation of the active role was carried out using the Antibiotic Stewardship Review and Approval Form, which was designed specifically for that purpose. It is filled and reviewed by the clinical pharmacist and infectious disease consultant. The form comprises 4 sections including (a) patient identification (name, gender, hospital number, location, and date), (b) the antibiotic requested (name, dose, route, frequency, duration), (c) the indication and management plan including whether it was prescribed empirically and whether appropriate investigations have been carried out, review plan, and diagnosis, and (d) the approval section including the MRP and Clinical Pharmacist identifications, and date of approval. [file 8140429.f1.zip › 8140429.f1/Boxes.docx]

# Boxes

**Box 1**. Content of the evidence-based empiric anti-infective use guidelines (EAIUG) implemented at the International Extended Care Centre (IECC), Jeddah, Saudi Arabia.

| **#** | **Care situation / guideline** | **#** | **Care situation / guideline** |
| --- | --- | --- | --- |
| 1 | Empiric Antibiotic Prescribing Guideline | 14 | Hospital-Acquired Infections in Adults |
| 2 | Empiric Antibiotic Use in case of Septic Shock | 15 | Central line associated Blood Stream Infection (CLABSI) |
| 3 | Risk factors for Multidrug Resistant Organisms or Fungemia. | 16 | Hospital-Acquired Pneumonia (HAP) & Ventilator Associated Pneumonia (VAP) |
| 4 | Community-acquired Infections in Adults | 17 | Hospital-acquired complicated Intra-Abdominal Infection (cIAI) |
| 5 | Endocarditis (Infective) | 18 | Management of Patients with Sepsis |
| 6 | Osteomyelitis | 19 | Surgical Antibiotics Prophylaxis Guideline |
| 7 | Diabetic Foot Infection | 20 | Antimicrobial Prophylaxis for Percutaneous Endoscopic Gastrostomy |
| 8 | Skin and Soft Tissue Infection | 21 | (PEG) Tube Insertion in Adults |
| 9 | Peritonitis | 22 | Antimicrobial Prophylaxis for Tracheostomy in Adults |
| 10 | Brucellosis | 23 | Switching IV antimicrobial to PO |
| 11 | Meningitis and Encephalitis (Bacterial) | 24 | Therapeutic Drug Monitoring for Aminoglycosides dosing |
| 12 | Brain Abscess | 25 | Therapeutic Drug Monitoring for Vancomycin |
| 13 | Genitourinary tract infection | 26 | Colistin dosing guidelines |

**Box 2**. Key performance indicators (KPIs) of the antimicrobial stewardship program – Definition and Scope.

| **KPI** | **Scope** | **Definition** |
| --- | --- | --- |
| **IV-to-oral switch** | Indicates the change in prescribing practice following the ASP implementation | Percentage of IV antibiotic prescriptions that were switched to the corresponding oral form in accordance with the specific EAIUG (sheet #23 in **Box 1**). In the present study, only five index antibiotics were analyzed (ciprofloxacin, levofloxacin, trimethoprim-sulfamethoxazole, and amoxicillin-clavulanate). |
| **Consumption of restricted IV antimicrobials** | 16 restricted IV antimicrobials included: moxifloxacin; levofloxacin; piperacillin; cefepime; colistin; meropenem; imipenem; caspofungin; tigecycline; micafungin; anidulafungin; voriconazole; amphotericin-B; linezolid; amikacin. | Consumption was defined and computed as the yearly number of consumed units (vials, boxes, ampoules, or bags) per antibiotic, divided by the corresponding number of patient-days. The overall consumption of restricted IV antimicrobial was calculated by pooling the consumed units for all antibiotics. |
| **Cost of restricted antibiotics** | Used as an indicator of the cost-effectiveness of ASP | The number of consumed units was multiplied by the unit cost for each drug, with respect to the applied prices. The cost was calculated and presented as 1,000 Saudi Riyal (kSAR) per 1,000 patient-days. Additionally, to adjust for yearly variation in drugs prices, adjusted (mean-standardized) costs were calculated using the average price during the six study years (2015-2020). |
| **Defined daily doses index** | Recommended by the World Health Organization (WHO) as standard measure of drug utilization enabling monitoring and comparative analysis of drug consumption across settings and countries.27 | defined as “the assumed average maintenance daily dose for a drug used for its main indication in adults”. It is calculated by dividing the actual dose of the drug by the corresponding drug-specific factor, as provided in the WHO official website.28  In the present study, yearly DDD was calculated per 1,000 patient-days. Only ten antibiotics (levofloxacin, piperacillin-tazocillline, cefepime, colistin, meropenem, imipenem, caspofungin, tigecycline, amikacin, and linezolid) were considered for this key performance indicator. Additionally, an overall DDD index was computed as the mean yearly DDD of all antibiotics by 1,000 patient-days. |
| **Antibiogram** | The antibiogram was monitored using semi-annual and annual cumulative reports of antimicrobial susceptibility rates of common microbial pathogens to antimicrobials available in the hospital formulary. It was intended to be used as a source to direct empiric antimicrobial therapy. In ASP, antibiogram was used to monitor the change over time of the antimicrobial susceptibility profile (sensitivity rate) of a given microbial pathogen. | for each year, the number of isolates for each pathogen was used as the denominator to calculate the percentage of isolates that were sensitive to each antimicrobial. Given the number of antimicrobials tested, the present study used the overall antimicrobial sensitivity index for each pathogen, which is an estimate of the overall antimicrobial sensitivity of a given pathogen and is calculated as the average sensitivity rate of the tested antimicrobials. Natural resistance was excluded from this analysis. |
| **Multidrug resistance in hospital-acquired infections (MDR-HAI)** | - | Defined as a pathogen being resistant to at least one agent in three or more antibiotic categories. The yearly number of MDR-HAI was expressed by 1,000 patient-days. |
